# Spatial heterogeneities in women’s adherence to sulfadoxine pyrimethamine prophylaxis in Tanzania: Findings from the 2022 TDHS–MIS

**DOI:** 10.1016/j.ijregi.2025.100795

**Published:** 2025-10-30

**Authors:** Mbwiga Sote Aloni

**Affiliations:** Department of Mathematics, Physics and Informatics, Mkwawa University College of Education, Iringa, Tanzania

**Keywords:** Tanzanian zone, SP/Fansidar, Spatial heterogeneities, Currently pregnant women, Malaria, TDHS-MIS

## Abstract

**Objectives:**

Most pregnant women in Tanzania do not adhere to the recommended use of SP/Fansidar for malaria prevention, posing a significant challenge to malaria control efforts. The study examines zonal heterogeneities in the uptake of SP/Fansidar doses in Tanzania.

**Methods:**

This cross-sectional study analyzed 2022 TDHS-MIS data to examine sociodemographic and geographic determinants of sufficient SP/Fansidar uptake among pregnant women in Tanzania.

**Results:**

Among 4157 women aged 15-49, those with secondary or higher education were 1.37 times more likely to take three or more SP/Fansidar doses, while women aged 35-39 were 34% less likely than those aged 15-19. SP/Fansidar doses uptake was higher in the Northern (adjusted odds ratio [aOR] = 1.78), Lake (aOR = 1.57), Southern (aOR = 1.44), and Eastern (aOR = 1.38) zones but 73% lower in Zanzibar (aOR = 0.27).

**Conclusions:**

Uptake of ≥3 SP/Fansidar doses among women in Tanzania remains generally low, with significant disparities across geographic zones and sociodemographic groups. The Northern, Southern, Lake, and Eastern zones show higher coverage, while Zanzibar lags substantially behind. Factors such as place of residence, education, maternal age, wealth status, and parity are key determinants, underscoring the need for carefully designed, geographically targeted interventions to improve SP/Fansidar adherence in the most affected zones.

## Highlights

• The study analyzed geographical disparities in SP/Fansidar uptake among pregnant women in Tanzania using 2022 TDHS-MIS data.
• A total of 4157 women aged 15-49 were included under a cross-sectional, two-stage stratified design.
• Zonal variations were found; uptake was higher in the Northern, Southern, Lake, and Eastern zones but lowest in Zanzibar.
• Residence, education, age, wealth, and parity significantly influenced uptake.
• The study underscores the need for targeted, zone-specific interventions to improve SP/Fansidar coverage.

## Introduction

Malaria is one of the most serious health threats worldwide, predominantly in tropical and subtropical regions. The current World Malaria Report from the World Health Organization (WHO) indicates that an estimated 2.2 billion cases and 12.7 million deaths from malaria have been averted worldwide since 2000 [1]. Malaria has remained a serious universal health hazard, particularly in the WHO African region. In 2023 alone, there were an estimated 263 million cases and about 597,000 malaria deaths worldwide [1]. This brings the total to about 11 million cases noted in 2022. Although the rate of malaria incidence has dropped over the last two decades due to improvements in malaria control interventions [2], some specific susceptible groups continue to face disproportionate threats. Pregnant women are especially vulnerable due to the physiological changes in pregnancy that reduce immunity and increase vulnerability to malaria infection [3]. It is estimated that malaria affects more than 32 million pregnancies, primarily in sub-Saharan Africa, where malaria transmission remains intense and perennial [4].

The consequences of malaria in pregnancy are both maternal and neonatal. Malaria in pregnant women may increase the risk of severe maternal complications such as anemia and adverse fetal outcomes, including miscarriage and maternal mortality [3]. Malaria in an infant is strongly associated with stillbirth, intrauterine growth restriction, low birth weight, preterm delivery, and increased neonatal death [5]. It is projected that malaria during pregnancy accounts for 10,000 maternal deaths and nearly 200,000 neonatal deaths per year [6]. This is one of the principal avoidable causes of adverse pregnancy outcomes in malaria-endemic regions. The WHO recommends intermittent preventive treatment in pregnancy (IPTp) with sulfadoxine-pyrimethamine (SP/Fansidar) to reduce the risk of malaria infection among pregnant women. Despite global efforts by the WHO, most women do not receive the recommended number of doses during pregnancy [7]. This challenge poses a substantial risk to most pregnant women who remain ineffectively secured against malaria.

According to the WHO guidelines for malaria [8], IPTp using *sulfadoxine-pyrimethamine* is recommended for all pregnant women residing in malaria-endemic regions where malaria transmission poses a significant risk. The regimen should begin in the second trimester, with doses given at least one month apart to ensure a minimum of three doses are received before delivery. This intervention aims to reduce maternal malaria infection, anemia, and adverse birth outcomes. However, this recommendation does not apply uniformly across all of sub-Saharan Africa, as malaria transmission intensity varies considerably between regions. According to the literature, only 35% of pregnant women in sub-Saharan Africa were reported to have received the recommended three doses of SP/Fansidar [9]. Despite WHO efforts to promote the use of SP/Fansidar, most pregnant women do not follow the recommended regimen of at least three doses during pregnancy, highlighting a significant implementation gap.

Despite the recent progress in malaria prevention in Tanzania, the epidemic has continued to pose a significant burden to the health sector. According to (2022), malaria prevalence among children aged 6-59 months was 8% in Tanzania. Malaria prevalence was reported to be higher in the Lake and Western zones and lower in the Southern zones. Less than 1% of children in Zanzibar tested positive for malaria [10]. Malaria in pregnant women remains a major contributor to maternal anemia, adverse birth outcomes, and neonatal mortality. Pregnant women are advised to take SP/Fansidar during routine antenatal care (ANC) visits, free of charge, starting in the second trimester. However, the uptake of the WHO recommendations of at least three doses in Tanzania has not reached the desired target. According to the current survey by NBS, OCGS, and ICF [10], 90% of women aged 15-49 with a live birth or stillbirth in the two years before the survey received ANC from a skilled provider, but less than one-third of pregnant women took the recommended three or more doses. This figure means that a large proportion of Tanzanian pregnant women still take fewer than the recommended doses, which undermines the country’s efforts to reduce malaria in pregnancy. Literature has identified multiple factors associated with SP/Fansidar uptake, including late initiation of ANC visits, limited number of ANC visits, stockouts of SP/Fansidar in health facilities, misconceptions about SP/Fansidar, parity, education, limited awareness of SP/Fansidar benefits, and social, cultural, and economic constraints as barriers to undertaking SP/Fansidar doses [7,11,12]. These barriers lead most pregnant women to receive fewer than the recommended three doses for optimal protection. However, there is limited evidence on how SP/Fansidar uptake varies across Tanzania's geographic zones (Western, Lake, Central, Eastern, Northern, Southern, and Zanzibar). Therefore, the current study examines spatial heterogeneity in SP/Fansidar uptake among currently pregnant women and evaluates sociodemographic factors that may contribute to these regional differences.

### Objective of the study

The present study examines spatial heterogeneity in the uptake of sufficient doses of SP/Fansidar among currently pregnant women aged 15-49 years across Tanzania's geographical zones.

## Method

### Study design

A cross-sectional study design was used to examine spatial heterogeneity in the use of sufficient SP/Fansidar dosage during pregnancy across different geographic zones of Tanzania. The study employed data from the 2022 Tanzania Demographic and Health Survey and Malaria Indicator Survey (2022 TDHS-MIS). The datasets were obtained through the Demographic and Health Survey (DHS) Program at https://microdata.nbs.go.tz/index.php/catalog/38/get-microdata.

### Study population

The population of the current study included women of reproductive age who participated in the 2022 TDHS-MIS and reported the number of SP/Fansidar doses taken during their current pregnancy. Thus, the study obtained a sample of 4157 women aged 15-49 years, selected from the dataset derived from the pregnancy and postnatal care recode file (TZNR81FL).

### Sampling and sample size

The study utilized data from the 2022 Tanzania Demographic and Health Survey and Malaria Indicator Survey (2022 TDHS-MIS). The DHS employed a two-stage stratified sampling design to produce nationally representative estimates for Tanzania Mainland and Zanzibar. In the first stage, clusters (enumeration areas) were selected from the 2012 Tanzania Population and Housing Census. In the second stage, 26 households per cluster were systematically selected. Women aged 15-49 who were usual residents or visitors in the selected households the night before the survey were eligible for interview. For this study, the sample was restricted to women who reported being pregnant during the survey, resulting in a total weighted sample of 4,802 currently pregnant women across Tanzania’s 31 regions. DHS sample weights (v005/1,000,000) were applied to correct for unequal selection probabilities and ensure representativeness.

### Study variables

The study examined the sufficiency of SP/Fansidar dosage during pregnancy as the outcome variable, categorized as insufficient coverage (coded “0” if pregnant women took fewer than three doses) and sufficient coverage (coded “1” if pregnant women took three or more doses). For computational purposes, the outcome variable was organized as follows:

$$\begin{array}{ccc} & & \text{Sufficieny}\;\;\text{of}\;\;\;\text{SP}/\text{Fansidar}\;\;\text{Dosage}\;\\ & & =\left\{ \begin{array}{c} 0\;\;\;\text{if}\;\;\text{Pregnant}\;\text{women}\;\text{took}\;\text{fewer}\;\text{than}\;\text{three}\;\text{doses}=\text{INSUFFICIENT}\;\;\;\text{COVERAGE}\\ 1\;\;\;\text{if}\;\;\text{Pregnant}\;\text{women}\;\text{took}\;\text{three}\;\text{or}\;\;\text{more}\;\;\;\text{doses}=\text{SUFFICIENT}\;\;\text{COVERAGE} \end{array}\right. \end{array}$$

The independent variables investigated (with their categories in brackets) were: (1) place of residence (urban and rural); (2) highest education level (no education, primary education, secondary and higher); (3) age of women (broken into categories of 5-years interval size starting from 15-19); (4) marital status (never married, married/cohabiting, and ever married [i.e., widowed or divorced]), (5) wealth index (poor, middle, and rich), (6) Tanzanian zone of residence: Western (comprising the regions of Tabora and Kigoma), Northern (Kilimanjaro, Tanga, and Arusha), Central (Dodoma, Singida, and Manyara), Southern (Iringa, Njombe, Ruvuma, Lindi, and Mtwara), Lake (Kagera, Mwanza, Geita, Mara, Simiyu, and Shinyanga), Eastern (Dar es Salaam, Pwani, and Morogoro), and Zanzibar (Kaskazini Unguja, Kusini Unguja, Mjini Magharibi, Kaskazini Pemba, and Kusini Pemba); (7) antenatal visit during pregnant (no and yes); (8) final say on women’s health care (women itself, women and her partner, and partner or someone else); and (9) birth in the last 5 years (no birth, 1, 2 and ≥3) as defined in the 2022 Tanzania Demographic and Health Survey and Malaria Indicator Survey Report [10].

### Data analysis

The study analyzed the doses of SP/Fansidar that a woman took during pregnancy among reproductive women aged 15-49. Data analysis was performed in STATA version 14.1 using survey procedures to account for the complex survey design, including clustering, stratification, and weighting. The analysis involved weighing the variables under study. However, the weighting resulted in an increase in the standard error measure, possibly due to the imputation of the non-response rate in the calculation of the estimates. The weighted binary analysis, using crude odds ratio (cOR), was conducted to examine the association between the number of times a woman took SP/Fansidar doses during pregnancy and each explanatory variable. Explanatory variables were selected for the multivariable logistic regression model to assess the effect of adjusted variables on the number of times a woman took SP/Fansidar doses during pregnancy when either it was significant (*P*-value <0.05) in the crude logistic regression model or was previously reported as statistically significant in earlier published studies.

## Results

### Univariate and bivariate analysis of study variables

The findings in Table 1 present the characteristics of the 4157 women. The data show that almost two-thirds (62.7%) of respondents reported having an insufficient dose of SP/Fansidar during their pregnancy. Respondents residing in rural areas constitute about 70%, and 56.5% had primary education. Concerning the age of respondents, those aged between 26-29 and 20-24 comprised the majority (25.5% vs 24.8%). Regarding marital status, married or cohabiting respondents comprised the majority (81.0%), while only 8.7% were never-married respondents.

**Table 1:** Univariate and bivariate analysis of the study variables.

Variable	Univariate analysis (Unweighted)		Bivariate analysis (Weighted)		
	Category	Frequency “n” percentage (%)	Crude odds ratio	95% confidence interval	*P*-value
Sufficiency of SP/Fansidar dosage during pregnancy	Insufficient	2608 (62.7)			
	Sufficient	1549 (37.3)			
Place of residence	Urban	1230 (29.6)	1		
	Rural	2927 (70.4)	0.69	0.59-0.83	0.000
Highest education level for respondent	No education	806 (19.4)	1		
	Primary	2349 (56.5)	1.36	1.12-1.66	0.002
	Secondary and higher	1002 (24.1)	1.85	1.46-2.32	0.000
Age in 5-year groups	15-19	318 (7.7)	1		
	20-24	1031 (24.8)	0.74	0.54-1.01	0.056
	26-29	1058 (25.5)	0.91	0.67-1.23	0.526
	30-34	804 (19.3)	0.72	0.52-0.98	0.038
	35-39	579 (13.9)	0.59	0.42-0.83	0.002
	40-44	295 (7.1)	0.63	0.43-0.92	0.017
	45-49	72 (1.7)	0.68	0.38-1.21	0.189
Marital status	Never married	362 (8.7)	1		
	Married/cohabiting	3368 (81.0)	0.91	0.70-1.18	0.474
	Ever married	427 (10.3)	0.85	0.61-1.19	0.330
Wealth index	Poor	1741 (41.8)	1		
	Middle	834 (20.1)	1.27	1.05-1.54	0.013
	Rich	1582 (38.1)	1.46	1.24-1.72	0.000
Tanzanian zone	Western	398 (9.6)	1		
	Northern	442 (10.6)	2.00	1.47-2.74	0.000
	Central	428 (10.3)	1.31	0.94-1.82	0.106
	Southern	1235 (29.7)	1.70	1.31-2.22	0.000
	Lake	1096 (26.4)	1.74	1.32-2.29	0.000
	Eastern	512 (12.3)	1.80	1.33-2.44	0.000
	Zanzibar	46 (1.1)	0.35	0.14-0.89	0.029
Antenatal visit during pregnant	No	489 (11.8)	1		
	Yes	3668 (88.2)	0.99	0.79-1.25	0.971
Final say on women health care	Women itself	606 (14.6)	1		
	Women and partner	1893 (45.5)	1.16	0.92-1.46	0.205
	Partner and someone else	1658 (39.9)	0.84	0.67-1.07	0.155
Birth in last 5 years	No birth	45 (1.1)	1		
	1	2364 (56.9)	2.62	1.27-5.41	0.009
	2	1534 (36.9)	1.79	0.86-3.70	0.118
	≥3	214 (5.1)	1.78	0.81-3.96	0.153

Regarding wealth status, the majority of respondents (41.8%) were poor, followed by 38.1% who were rich, and those in the middle class comprised the lowest percentage (20.1%). Regarding residents in the Tanzanian zone, it was observed that the Southern and Lake zones accounted for the majority of respondents, with 29.7% and 26.4% of the total respondents, respectively. It was observed that more than 88% of respondents attended antenatal visits during pregnancy. The majority of respondents (45.5%) said the final say on women's health care was with women and their partners. Among respondents who gave birth in the last 5 years, about 57% had two births, and only 1.1% had none.

The bivariate analysis between the dependent variable “sufficient dosage of SP/Fansidar during pregnancy” and each explanatory variable was analyzed using crude logistic regression models; the results are presented as cOR, 95% confidence intervals, and *P*-values, as shown in Table 1. The results shows that women with primary education (cOR = 1.36, *P* = 0.002), women with secondary education and higher (cOR = 1.85, *P* = 0.000), women with middle income (cOR = 1.27, *P* = 0.013), women with rich income (cOR = 1.46, *P* = 0.000), women residing in northern regions of Tanzania (cOR = 2.00, *P* = 0.000), women living in the southern part of Tanzania (cOR = 1.70, *P* = 0.000), women residing in lake regions of Tanzania (cOR = 1.74, *P* = 0.000), women residing in the eastern areas of Tanzania (cOR = 1.80, *P* = 0.000), and women with only one birth in the last 5 years preceding the 2022 TDHS-MIS (cOR = 2.62, *P* = 0.009) were statistically significantly positively associated with taking a sufficient dosage of SP/Fansidar during pregnancy. Moreover, women residing in rural areas of Tanzania were 31% less likely to take an adequate dose of SP/Fansidar during pregnancy than those residing in urban areas (cOR = 0.69, *P* = 0.000). Regarding the age of women, those aged 15-19 were used as the reference category; the odds of women who took a sufficient dosage of SP/Fansidar were 28% less likely among women aged 30-34 (cOR = 0.72, *P* = 0.038), 41% less likely among women aged 35-39 (cOR = 0.59, *P* = 0.002), and 37% less likely among women aged 40-44 (cOR = 0.63, *P* = 0.017). Further evidence of a decreased likelihood of taking a sufficient dosage of SP/Fansidar was observed among respondents residing in Zanzibar, such that women who reported taking an adequate dosage of SP/Fansidar during pregnancy were 65% less likely than women residing in the western zone of Tanzania (cOR = 0.35, *P* = 0.029).

### Multivariable logistic regression model of sufficient uptake of SP/Fansidar dosage during pregnancy

Results from the multivariable random-effects logistic regression model (adjusted) for factors associated with sufficient SP/Fansidar dosage during pregnancy are presented in Table 2. Findings reveal that the higher the level of education, the higher the likelihood of women taking SP/Fansidar during pregnancy, such that women with secondary or higher education had a 37% higher chance of taking a sufficient dose of SP/Fansidar compared to women without education (adjusted odds ratio [aOR] = 1.37, *P* = 0.017). On the other hand, the number of SP/Fansidar doses taken during pregnancy was 34% lower among women aged 35-39 than among women aged 15-19 (aOR = 0.66, *P* = 0.017). In term of women’s zone of residence in Tanzanian, the likelihood that women took a sufficient dosage of SP/Fansidar during pregnancy was significantly 78%, 44%, 57%, and 38% higher among women from the northern zone (aOR = 1.78, *P* = 0.000), southern zone (aOR = 1.44, *P* = 0.008), lake zone (aOR = 1.57, *P* = 0.001), and eastern zone (aOR = 1.38, *P* = 0.045), respectively, compared to women from the western zone. On the other hand, the odds ratio for women taking a sufficient dose of SP/Fansidar during pregnancy was 73% lower among residents of Zanzibar zones than among residents of the western zone of Tanzania (aOR = 0.27, *P* = 0.007). Among women who gave birth in the 5 years preceding the 2022 TDHS-MIS, the likelihood of taking a sufficient dose of SP/Fansidar during pregnancy was 2.5 times higher among women with one birth than among those without a birth during the last 5 years.

**Table 2:** Multivariate logistic regression model of sufficient uptake of SP/Fansidar dosage during pregnancy.

Variable	Category	Adjusted odds ratio	95% confidence interval	*P*-value
Place of residence	Urban	1		
	Rural	0.82	0.65-1.03	0.081
Highest education level for respondent	No education	1		
	Primary	1.19	0.97-1.47	0.084
	Secondary or higher	1.37	1.06-1.78	0.017
Age in 5-year groups	15-19	1		
	20-24	0.79	0.58-1.08	0.151
	26-29	0.94	0.68-1.29	0.707
	30-34	0.78	0.57-1.08	0.132
	35-39	0.66	0.47-0.92	0.017
	40-44	0.70	0.48-1.03	0.072
	45-49	0.78	0.43-1.41	0.409
Wealth index	Poor	1		
	Middle	1.12	0.92-1.37	0.265
	Rich	1.07	0.85-1.34	0.564
Tanzanian zone	Western	1		
	Northern	1.78	1.29-2.46	0.000
	Central	1.25	0.89-1.73	0.185
	Southern	1.44	1.10-1.90	0.008
	Lake	1.57	1.19-2.07	0.001
	Eastern	1.38	1.01-1.89	0.045
	Zanzibar	0.27	0.10-0.69	0.007
Birth in last 5 years (parity)	No birth	1		
	1	2.50	1.22-5.12	0.012
	2	1.84	0.89-3.78	0.099
	≥3	1.87	0.84-4.16	0.122

## Discussion

The study findings reveal that fewer than 38% of pregnant women received three or more doses of SP/Fansidar, as recommended by WHO guidelines for malaria prevention and control during pregnancy. These data corresponded to fewer than two in every five pregnant women who adhered to the recommendation. The 37.3% observed in this study is higher than the 13% reported in the 2022 Kenya Demographic and Health Survey [13] and lower than the 56% reported in the 2022 Uganda Demographic and Health Survey [14]. The study findings imply that a proportion of pregnant women and their unborn children in Tanzania are at risk of malaria-related effects, as many do not receive sufficient doses of SP/Fansidar. Having secondary or higher education was positively associated with women receiving three or more doses of SP/Fansidar during pregnancy. The observed findings may have occurred because educated women are more likely to understand and adhere to health messages delivered through various media and during ANC visits, particularly regarding the dangers of malaria to pregnant women. Education empowers women by providing greater autonomy and self-confidence in making health decisions [15,16]. A recent study in Uganda found that uptake of IPTp was significantly associated with individual educational level [[17], [18], [19], [20]]. Women aged 35-39 were significantly less likely to take three or more doses of SP/Fansidar than those aged 15-19. A history of multiple pregnancies may create a sense of confidence and lower perceived susceptibility to malaria, which may reduce motivation to complete the recommended doses and limit antenatal visits. In contrast, younger pregnant women may adhere more closely to malaria prevention recommendations, possibly because they are in their prime reproductive years, attend ANC more regularly, and are more attentive to health messages. The current study’s findings differ from those reported in previous studies [19]. Their analysis showed that pregnant women aged 15-24 years were less likely to take optimal SP doses than those aged 36-39. The variation may be due to differences in the categorization of the age groups. In the current study, the youngest age group was defined as 15-19 years, whereas the former study categorized the age group as 15-24, which may exhibit distinct health-seeking behavior.

Regarding the Tanzanian zone of residence, women from the northern, southern, Lake, and eastern zones had a positive association with taking SP/Fansidar doses three or more times during pregnancy. The positive association may have been due to several factors, including strong access to health infrastructure and the effectiveness of malaria prevention programs. However, barriers to healthcare access among vulnerable groups highlight issues related to availability, affordability, and acceptability in public health facilities [21]. Other studies in Tanzania have also reported substantial variations in access to healthcare services, particularly between rural and urban areas, which influence the uptake of preventive interventions such as IPTp-SP [22,23]. Moreover, women residing in Zanzibar were 73% less likely to take SP/Fansidar doses three or more times than pregnant women living in the western zone. The lower uptake in Zanzibar may have been due to the zone’s relatively low malaria transmission, in which pregnant women may perceive the risk of malaria infection as minimal. While this study observed a significant association between the outcome variable and women’s zone of residence [19], the study [19] reported no significant relationship between these two variables. The variation in significance could be attributed to the scope of these studies. While the current research encompassed all Tanzanian zones of residence, the former study focused only on a specific ecological zone in Ghana. This may have narrowed the variability and power of the zone of residence on the outcome variable.

## Conclusion

The percentage of women who take three or more doses of SP/Fansidar during pregnancy is low in Tanzania. Aspects such as the highest education level attained, age, Tanzanian zone of residence, and women born in the last 5 years significantly predicted the number of times pregnant women took SP/Fansidar during pregnancy. The study findings highlight marked Tanzanian zone disparities in the uptake of malaria preventive treatment in pregnancy, with the northern, southern, Lake, and eastern zones performing better. Zanzibar zones have shown significant gaps that demand interventions. Awareness of the effects of these aspects on the outcome variable is crucial to the success of the country's antimalarial drug policy. The study recommends that the Ministry of Health, the National Malaria Control Programme, and other relevant stakeholders consider the effects of geographic zones when planning and implementing interventions to increase SP/Fansidar doses among pregnant women.

## Declaration of competing interest

The authors have no competing interests to declare.
